# Microbial degradation and community structure analysis of hydroxyl-terminated polybutadiene (HTPB)

**DOI:** 10.1186/s13568-021-01334-1

**Published:** 2021-12-27

**Authors:** Ying Zhang, Min Zou, Adil Farooq Lodhi, Yu-lin Deng

**Affiliations:** 1 School of Life Science, Beijing Institute of Technology, Beijing, 100081 China; 2 Department of Microbiology, Faculty of Biological Sciences, Hazara University, Mansehra, Pakistan

**Keywords:** HTPB, Microorganisms, Degradation, Microbial community structure

## Abstract

Hydroxyl-terminated polybutadiene (HTPB) is a curing adhesive that is commonly used in the production of ammunition, and it emerged during the time of war. After entering the peaceful era, several countries around the globe have focused on the destruction of expired ammunition using safe and economical methods in terms of consumption of energy. Microorganisms exhibit a highly efficient and environment friendly degradation capability for variety of refractory substances. Therefore, in this study we screened five strains of microorganisms from five environmental soil samples for their ability to degrade HTPB. These microorganisms were identified as *Microbacterium trichothecenolyticum*, *Microbacterium esteraromaticum*, *Arthrobacter pascens*, *Pseudonocardia carboxydivorans* and *Ochrobactrum anthropic* based on 16S rRNA gene similarity index. We observed the uncorroded and corroded HTPB sample through scanning electron microscopy and observed the formation of lot of holes and gullies in HTPB after corrosion. An 18S rRNA gene clone library was constructed for HTPB-degrading fungi. Based on the results of library evaluation, it was found that the structure of the HTPB-degrading fungi community was relatively simple. A total of 54 positive clones were obtained. These clones represented some uncultured microorganisms that were closely related to *Scytalidium lignicola*, *Pseudokahliella* and *Gonostomum strenuum*. This study will help in the implementation of environment friendly degradation strategies for HTPB degradation.

**Supplementary Information:**

The online version contains supplementary material available at 10.1186/s13568-021-01334-1.

## Key points

Whether HTPB can be safely and greenly processed in peaceful times has attracted much attention. This research aims to degrade HTPB by microorganisms and screen out cultivable bacteria. A clone library was constructed for HTPB-degrading fungi.

## Introduction

Hydroxyl-terminated polybutadiene (HTPB) is a telechelic polyol prepolymer that reacts with a crosslinker and a chain extender to form a cured product with a three-dimensional network structure. It is one of the most commonly used polymers in composite solid propellants, so it has a wide range of applications in military and aerospace fields (Arisawa and Brill 1996; Toosi et al. 2015; Chaturvedi and Dave 2019; Ju et al. 2020; Wu et al. 2021). There are a number of long-term ammunition storage facilities present around the world that are no longer usable and must be destroyed (Wu et al. 2021). The ordinary explosive destruction process not only requires a lot of manpower and material resources, but it is also quite dangerous, besides causing huge energy waste and environmental pollution (Celina et al. 2002; Keković 2011; Junsheng et al. 2015). HTPB binder can be used as a solid fuel in hybrid rockets in the aerospace field to provide a higher solid propulsion specific impulse. However, carbon black that can cause potential climate change is produced during its use. Rocket engines emit more carbon black per unit mass of propellant than airplanes (DeLuca et al. 2013).

The current research on the degradation of HTPB mainly focuses on the use of thermal degradation, but thermal degradation requires quite high temperature. In the temperature range of 160–250° C, only the carbon–carbon double bonds on the main chain of HTPB can be broken, and a temperature above 350° C is required for complete decomposition (Ganesh et al. 2000; Chatragadda and Vargeese 2017). Therefore, thermal degradation is not the most economical, effective and environmentally friendly method for processing HTPB waste (Ganesh et al. 2000).

Since Kanavel first observed the biodegradability of polyurethane (Wu et al. 2021), the ability of microorganisms to degrade various polymeric materials has received a lot of attention and this ability has been utilized in various applications. The use of microorganisms for degradation and treatment of polymeric compounds not only consumes less energy, but the by-products produced by decomposition can also be used by microorganisms. In this way, the process of recycling and reuse of compounds becomes easy. Wu Kai et al. through experiments screened out two bacteria that can use the HTPB/TDI binder system as the sole carbon source, *Coccobacillus* sp. and *Arthrobacter* sp. (Wu et al. 2021).

If community structure of HTPB degrading microorganisms can be analyzed, it would become easy to study their growth requirements. In this way, it would be possible to design a culture medium having necessary growth requirements of HTPB degrading microorganisms. It would not only result in successful isolation of HTPB degrading microorganisms but also environment friendly degradation of HTPB materials would be possible. Research has shown that the microbial diversity obtained through conventional culture-based techniques only accounts for 0.1–5% of the total diversity (Amann et al. 1995; Blagodatskaya and Kuzyakov 2013). Therefore, culture independent methods are more conducive in providing a comprehensive reflection of the microbial community that degrades HTPB materials. In this study, pure HTPB (M_w_ = 2800 g mol^−1^) (Vidal et al. 2004) used to make ammunition was subjected to degradation, and the microorganisms obtained from five soil samples were used for the purpose of its degradation. The efficiency of microbial community present in those soil samples in terms of HTPB degradation was separately evaluated for each sample. The community structure of HTPB degrading microorganism is relatively simple, we used both culture based and culture independent methods to analyze one of the most efficient HTBP degrading soil microbial communities. These methods were used to explore the structure and composition of such a microbial community. Besides, the application of scanning electron microscopy also helped to explore the surface morphology characteristics of HTPB after degradation by microorganisms.

## Materials and methods

### Cultivation of HTPB microflora

The HTPB material was cut into blocks having the dimensions, 1 cm length and 0.3 cm height. This material was buried in the soil 10 cm deep on the same vertical line. The buried location and soil type were the loess type of Baoding City, Hebei Province, the black soil type of Guangyuan City, Sichuan Province, the laterite type of Jinjiang Town, Chengmai County, Hainan Province, the laterite type wetland area of Xiamen City, Fujian Province, and the cinnamon soil type of Haidian District, Beijing.

After about 28–40 days, small pieces of HTPB were taken out along with 5 g of soil taken from 1.5 cm^2^ area of the surroundings. These were placed in a 50 mL sterile Corning centrifuge tube, sealed, and stored for further experimental procedures.

For the process of culturing, enrichment culture was used. For this purpose, small HTPB pieces and the surrounding soil (5 g) was added to a vitamin-free mineral liquid medium (Akutsu-Shigeno et al. 2006) (medium components included KH_2_PO_4_, 2.0 g/L; K_2_HPO_4_, 7.0 g/L) ; NH_4_NO_3_, 1.0 g/L; MgSO_4_, 0.1 g/L; FeSO_4_·7H_2_O, 10 mg/L; ZnSO_4_·7H_2_O, 1.0 mg/L; CuSO_4_,0.01 mg/L; MnSO_4_·6H_2_O, 2.0 mg/L), The incubation of enrichment culture was carried out at 28° C and 120 rpm. Once the microbial films were formed and observed on the surface of medium, the transfer of cultures was performed onto original medium having already added vitamins (Akutsu-Shigeno et al. 2006) (Nicotinamide 10.0 mg/L; pantothenic acid 2.5 mg/L; thiamine 2.5 mg/L; riboflavin 1.25 mg/L; pyridoxal 0.75 mg/L; *p*-aminobenzoic acid 0.6 mg/L; folic acid 0.5 mg/L; and biotin 0.1 mg/L, filtered and sterilized for later use).

### Microbial degradation of HTPB surface characteristics

The changes on surface of HTPB surface that occurred because of microbial degradation were observed using the following parameters:

#### Electron microscope observation of microbial attachment state on HTPB surface

For scanning electron microscopy, the HTPB in the liquid culture medium was cut into a small size of 0.5 cm × 0.5 cm × 0.15 cm, and 3% glutaraldehyde was used for the pretreatment of sample. This was done to facilitate the observation process. An ion sputterer (HITACHI MC1000, Japan) was used to spray gold, and then a scanning electron microscope (HITACHI SU8010, Japan) was used for observation.

#### Observation of corrosion of the surface of HTPB after removing the bacterial growth through electron microscope

Sterile tweezers were used to carefully take out the small pieces of HTPB from the culture medium, these were cut into samples with a size of 0.5 cm × 0.5 cm × 0.15 cm. Then bacterial biofilms were scrapped off from the surface. Then scanning electron microscope (HITACHI S-5500, Japan) was used to observe the corrosion of microbes on the surface of small pieces of HTPB.

### Study on the structure of microbial community that degrades HTPB

A detailed analysis of microbial community involved in the degradation of HTPB was performed using the following process:

#### Isolation, purification, and identification of cultivable microorganisms with HTPB degradation ability

When bacteria growing on the inner wall of the conical flask had formed a film that was clearly seen with the naked eye, along with increase in the turbidity of medium, it was a clear indication that bacteria capable of degrading HTPB could be screened further, grown, purified, and identified for future use.

A solid mineral salt medium having HTPB as the sole carbon source was first prepared using the same composition as mentioned above for liquid mineral medium except for the addition of agar (15%) before autoclave. The sterilized medium was poured in petri plates. Then small pieces of HTPB having dimensions, 0.5 cm × 0.5 cm × 0.15 cm, were placed on the surface of the culture medium.

On these petri plates two types of microorganisms were cultured. One type of culture contained microorganisms scrapped from the surface of small HTPB pieces, and the other type was cultured from the microbial biofilm formed on the inner walls of the culture bottle where the liquid medium was in direct contact with the air, and the film was clearly seen with the naked eye. These two types of microorganisms were streaked on culture plates and the plates were kept in incubator at 37° C. Further subculturing was performed for the separation and purification of microorganisms in the form of isolated colonies.

Microorganisms were initially classified on the basis of colony morphology (Deanna and Sutton 1999; Byzov et al. 2007; Sintchenko et al. 2007; Freiwald and Sauer 2009; Boyandin et al. 2013; Huang and Wu 2018), The 16-S rRNA gene sequence was then used to identify the isolated and purified bacterial cultures. TIANamp Bacteria DNA Kit (China, TIANGEN) was used to extract the purified bacterial DNA. Using the extracted bacterial DNA as a template, and P_0_ (5′-GAGAGTTTGATCCTGGCCAG-3′) and P_6_ (5′-CTACGGCTACCTTGTTACGA-3′) as primers, amplification of 16S rRNA gene was done. The amplification system is shown in Additional file 1: Table S1.

$${\text{PCR amplification conditions}}: 95\,^\circ {\text{C}}(5{\text{min}})-[95\,^\circ {\text{C}}(30{\text{S}})-56\,^\circ {\text{C}}(30{\text{S}})-72\,^\circ {\text{C}}(90{\text{S}})](25{\text{cycles}})-72\,^\circ {\text{C}}(10{\text{min}})-4\,^\circ{\text{C}}.$$

The PCR products were sent to Shenggong Bioengineering (Shanghai) Co., Ltd. for sequencing, and the pure, aligned sequences were analyzed using the National Center for Biological Information (NCBI) https://www.ncbi.nlm.nih.gov/BLAST program for the purpose of identification on the basis of 16S rRNA gene similarity. The neighbor-joining method of MEGA 6.0.6 software was used to construct a phylogenetic tree of the identified strains and the dominant strains, besides performing their phylogenetic analysis. The Kimura’2-parameter model was used to construct the phylogenetic tree, and the Bootstraping method was used to evaluate the phylogenetic tree. After every 1000 replications, when the Boostrap value of the strain would be greater than 50%, the strain will appear at the node of the phylogenetic tree.

#### Construction of molecular cloning library of HTPB degrading microflora

The culture medium for enrichment culture was centrifuged, the precipitate was preserved and then vacuum dried. The E.Z.N.A.™ Soil DNA Kit (Omega Biotek, Doraville, GA, USA) was used to extract the total DNA of the precipitated microorganisms. The 18S rRNA gene amplification was performed using ITS1 and ITS4 primers (Bellemain et al. 2010). The amplified target gene was purified and recovered using a common agarose gel DNA recovery kit (China, TIANGEN, DP209).

The recovered fungal-specific DNA band was ligated with pGEM-T Easy cloning vector (China, TIANGEN, VT302-01) at 4° C overnight. The ligation product could be used for the next transformation after 16 h. The recombinant vector was then transformed into the competent *E. coli* (TIANGEN, *E. coli* DH5α, China) cells and then incubated for 45 min.

100 µL of bacterial solution was taken and spread on the Ampicillin resistant (concentration 50 µg/mL) LB solid medium plate containing 16 µL of 0.1 mol/L IPTG and 40 µL of 0.05 g/mL X-Gal (Peptone 10 g/L, yeast extract 5 g/L, NaCl 10 g/L, agar 15 g/L). After overnight incubation at 37° C, white colonies were selected, and colony PCR was performed on the plasmid containing the 18S rRNA gene insert using T7 and SP6 universal primers. The samples were handed over to Shanghai Shenggong Company for sequencing.

## Results

### Observation of the surface of HTPB sample with bacterial membrane attached

**Fig. 1:**
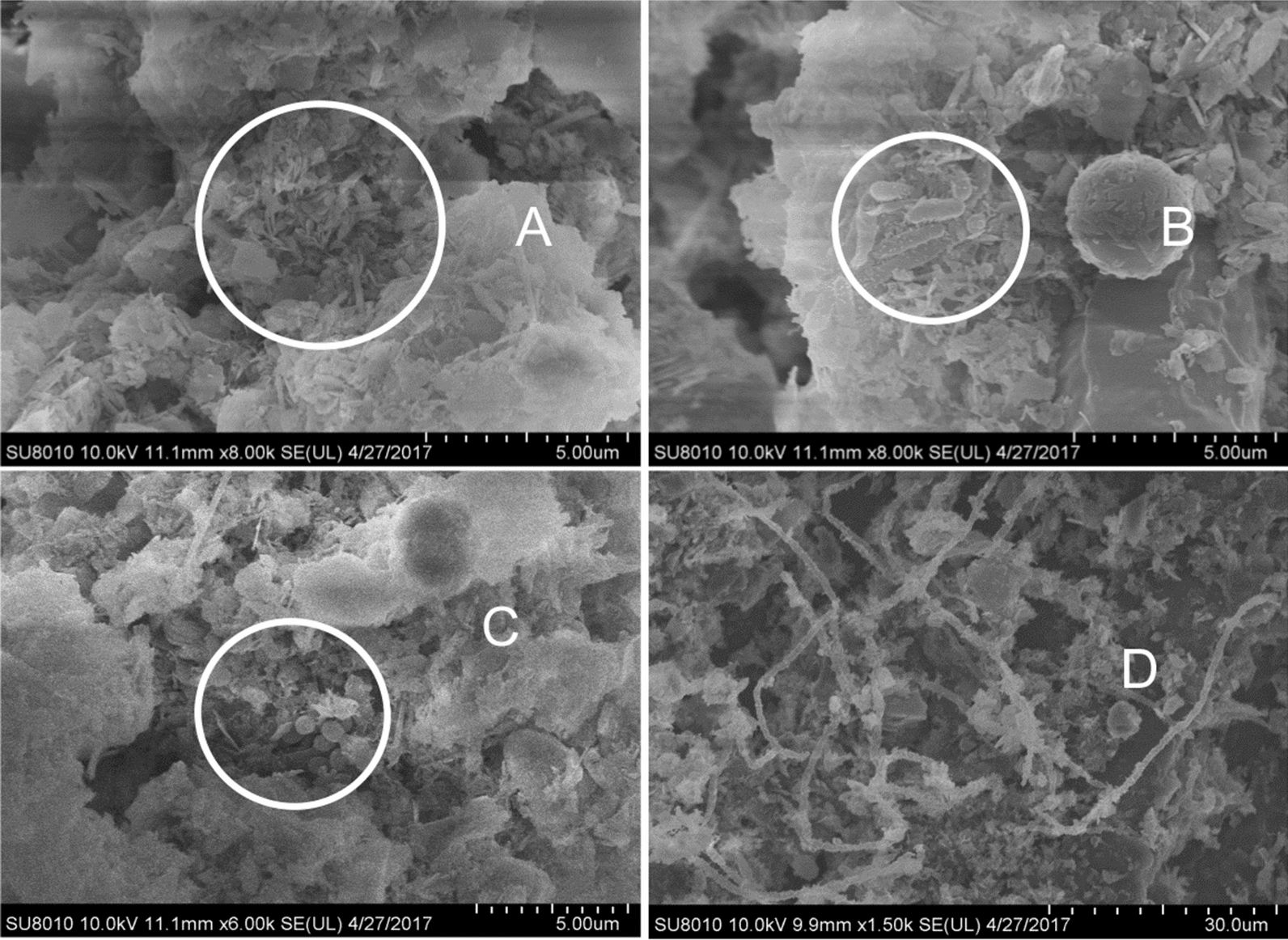
Scanning electron microscope observation of the surface and internal structure of HTPB sample with bacterial membrane attached (Pictures **A** and **B** show the rod-shaped bacteria in corroded HTPB material. **C** Shows the corrosive cocci in the HTPB material. **D** Shows the hypha structure cross-linked with HTPB)

It was observed by using scanning electron microscope that after a long incubation period, clearly pores and gullies were produced on the surface of HTPB. It happened under the action of microorganisms and a large number of rod-shaped bacteria and cocci were seen attached to these gully and pores (Fig. 1A–C). In addition, a large number of hyphae were also observed to penetrate into the HTPB (Fig. 1D). It is speculated that these microorganisms are directly involved in the degradation of HTPB materials.

**Fig. 2:**
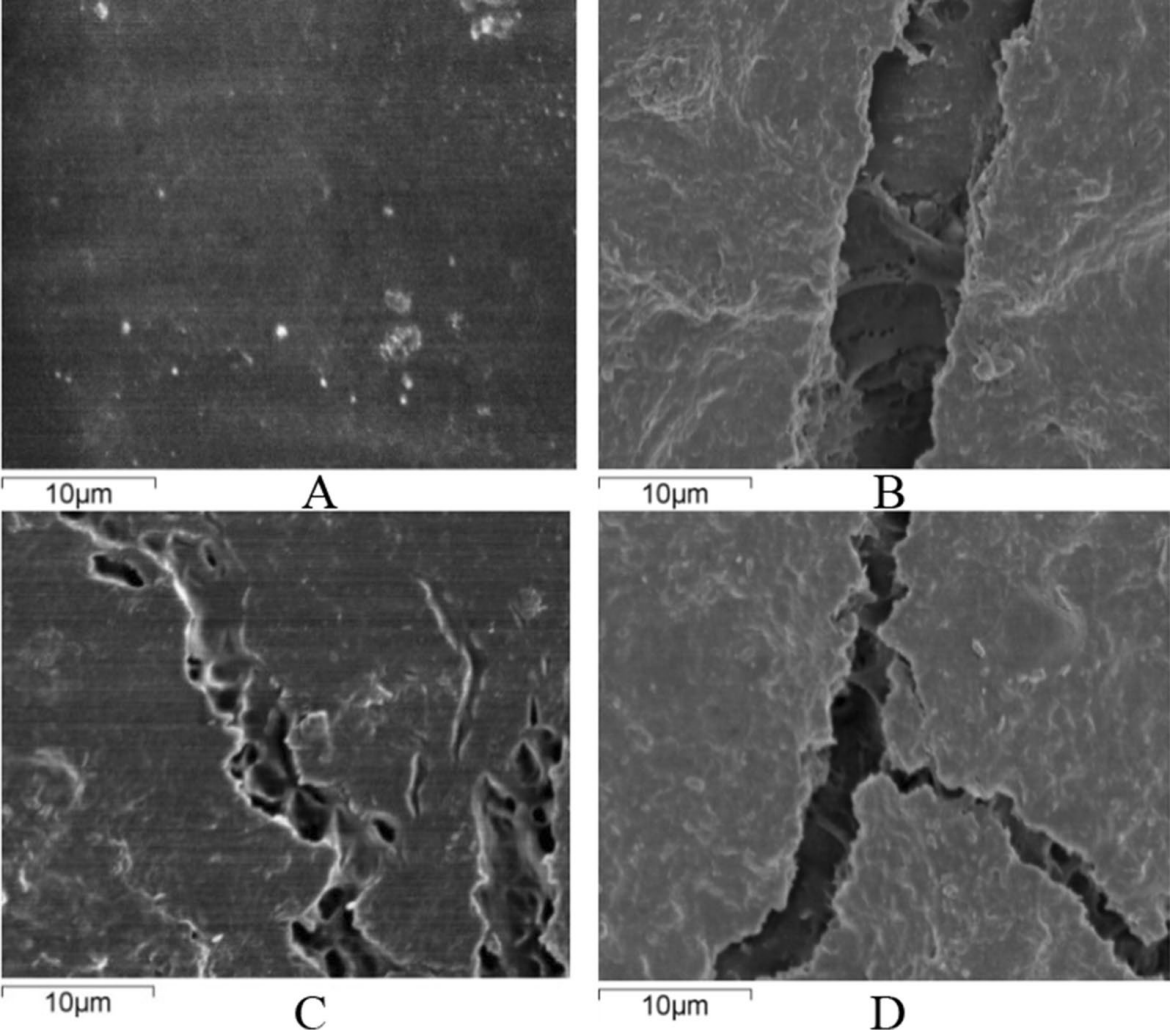
Scanning electron microscopy to observe the surface of the HTPB sample with the bacterial membranes removed (**A** shows the uncorroded HTPB material. **B**–**D** Show the corrosion effects on the surface of the HTPB material after microbial corrosion)

It can be seen from Fig. 2A that the surface of the uncorroded HTPB material is very flat, and there are no gullies or holes except for a few bubbles. After the action of microorganisms, the HTPB material obviously produced a lot of gullies and holes (Fig. 2B–D). Comparing the electron micrographs of the untreated HTPB material and the HTPB material that has undergone the action of microorganisms, it can be seen that the microorganisms have obviously destroyed the surface structure of HTPB.

### Cultivation and identification of HTPB degrading bacteria

**Fig. 3:**
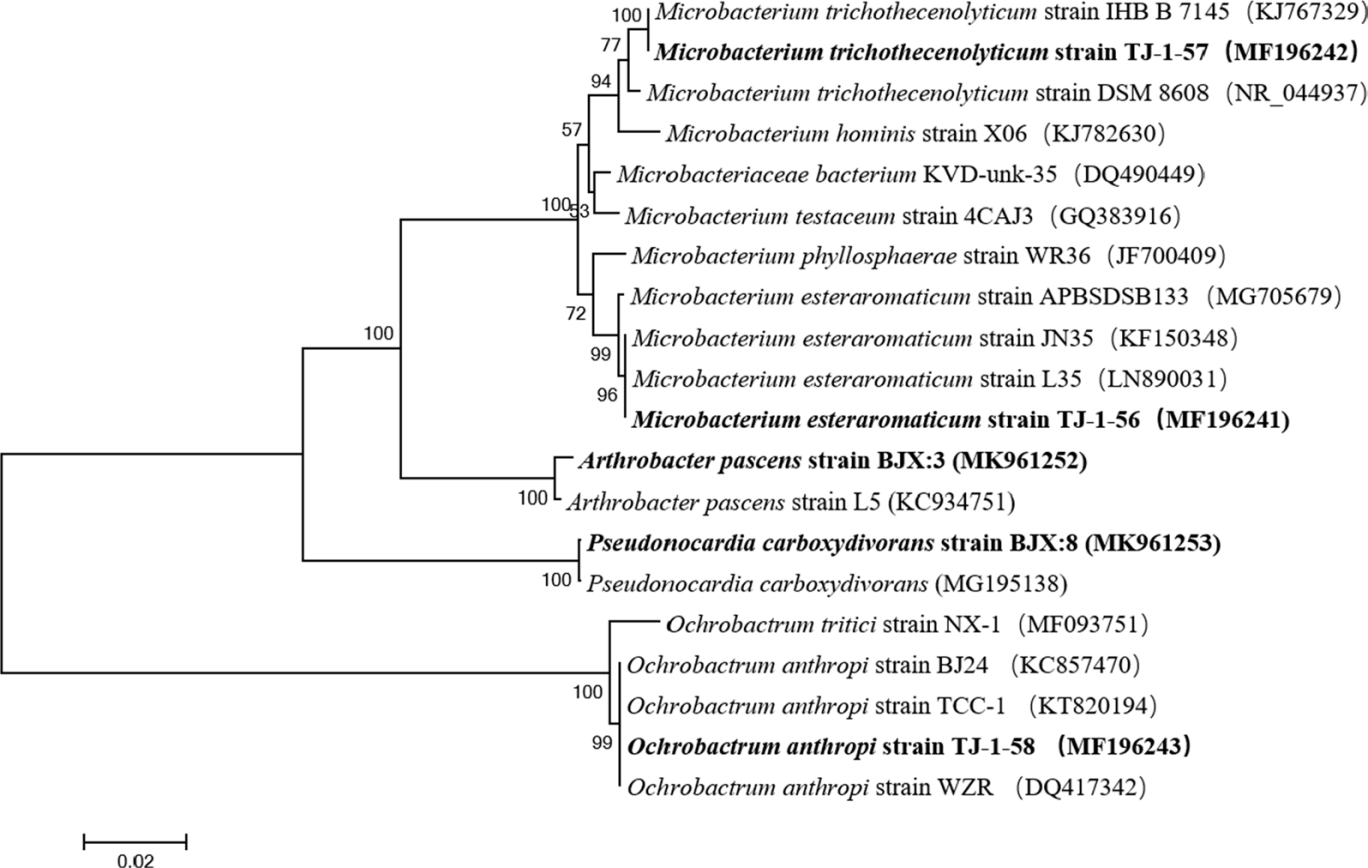
Phylogenetic tree of cultivable microorganisms

The sample of HTPB buried in soil was enriched and cultured in a mineral liquid medium, and then 5 strains of HTPB degrading bacteria were isolated by using vitamin-added oligotrophic medium with HTPB as the sole carbon source. After the 16S rRNA gene sequence of the isolated and purified bacteria was determined, and compared with the GenBank database, the phylogenetic tree of the five bacteria as shown in Fig. 3 was constructed.

From the phylogenetic tree of cultivable microorganisms in Fig. 3, it can be seen that the identified strains belong to *Microbacterium trichothecenolyticum*, *Microbacterium esteraromaticum*, *Arthrobacter pascens*, *Pseudonocardia carboxydivorans*, and *Ochrobactrum anthropi*.

### Construction of cloning library of HTPB degrading fungi

The results of the cloning library showed that a total of 54 clones were generated by cloning the 18-S rRNA gene region. Among them, the number of one clone genotype was 6, and the number of two clone genotype was 1. The statistical values of the library were as follows. The 18-S rRNA clone library of degrading bacteria contained 11 genotypes. The coverage value of the library was 88.89%, the Shannon index (H’) was 1.726, the richness (R) 72, and the uniformity (E) was 0.404. The above data proved that the storage capacity of the constructed clone library was sufficient to reflect the diversity of microorganisms in the actual sample.

**Fig. 4:**
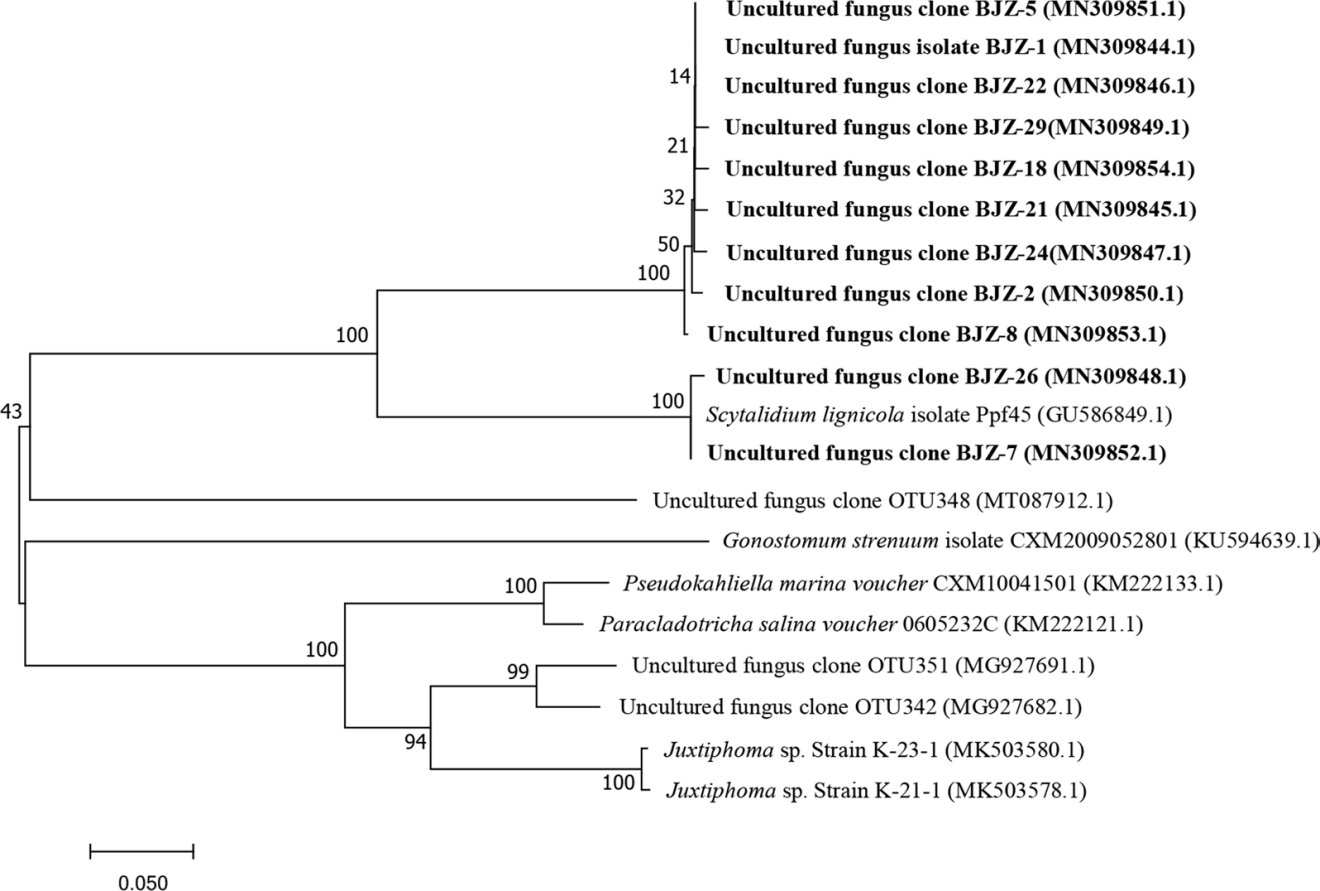
Phylogenetic tree of HTPB degrading fungi constructed by molecular clone library

Through careful analysis of the characteristics of the HTPB-degrading fungi community from the clone library, it was revealed that all the identified fungi were non-cultured microorganisms, and their phylogenetic status was closest to that of *Scytalidium lignicola*, *Pseudokahliella* and *G. strenuuum* (As shown in Fig. 4).

## Discussion

At present, the biodegradable HTPB material is in line with the concept of “green ammunition” and biodegradation is becoming the safest method of degradation. Therefore, this article explores the biodegradation capability and corrosion effects of the HTPB materials. Five types of bacteria were isolated and identified by conventional culturing methods, namely *Microbacterium esteraromaticum* strain TJ-1-56, *Microbacterium trichothecenolyticum* strain TJ-1-57, *Ochrobactrum anthropi* strain TJ-1-58, *Arthrobacter pascens* strain BJX:3 and *Pseudonocardia carboxydivorans* strain BJX: 8. Studies have shown that the continuous use of small HTPB as the sole carbon source in culture media can better domesticate and adapt microorganisms having HTPB degradation ability to grow on petri plates (Wu et al. 2021). The above five strains of bacteria mentioned in this study can all grow well on the medium with small pieces of HTPB as the sole carbon source. It has been concluded after observation through the electron microscope and after comparison of the HTPB material before and after the treatment with microorganisms. The surface of the treated HTPB material was observed by scanning electron microscope and many cracks and holes were observed, which proved that HTPB was indeed corroded by microorganisms. The strains identified in this study possessed a good degradation capability against the HTPB. Therefore, the community structure characteristics of HTPB-degrading microorganisms present in the soil environment were determined.

Since the characteristics of the fungal community that helped in the degradation of HTPB in the sample could not be evaluated by traditional pure isolation and culture methods; therefore, we used an 18S rRNA gene cloning library method that does not rely on isolation and culturing of microorganisms for their study. The results of the library showed that the diversity of fungi was low, and there was no need to use third-generation sequencing technology to explore the composition of the population. The results of the cloning library showed that the closely related HTPB-degrading fungi were mostly un-cultured clones and consisted up of three microorganisms, *Scytalidium lignicola*, *Pseudokahliella* and *Gonostomum strenuum*. According to reports, *Scytalidium lignicola* has the ability to decompose cellulose in nature, and it also has great potential in biodegrading phenolic compounds, cyanide and synthetic dyes, as well as degrading polycyclic aromatic hydrocarbons (Fukasawa et al. 2011). *Pseudokahliella* is abundant in soil, fresh water, and sea water, and can efficiently and rapidly degrade hydrophobic and refractory organic pollutants such as alkanes, aromatic hydrocarbons, and halogenated aromatic hydrocarbons.

At the same time, the scanning electron microscope results of this study clearly showed that the hyphae of these non-culturable fungi penetrated deep into the HTPB to participate in the process of degradation.

The biodegradation of HTPB is very important to solve the problem of long-term storage of ammunition, since the traditional destruction methods not only possess a high environmental impact, but also result in a huge waste of energy. The understanding of the diversity of HTPB-degrading microbial strains will contribute towards the realization of large-scale biodegradation of HTPB in the future. The results of this study indicate that HTPB-degrading bacteria are not only diverse, but are also widespread in the natural environment, affecting HTPB degradation in the environment. In future studies, microorganisms that can degrade HTPB can be screened from diverse soil environments, and such HTPB-degrading fungi and bacteria can be separated and grown in pure cultures by optimizing the culture medium. It can facilitate the understanding of mechanisms involved in the degradation of HTPB. In future, such studies can result in the provision of microbial resources for the effective degradation of HTPB in the future. At the same time, pretreatment of HTPB can be considered to make the degradation of such substances by microorganisms more effective.

## Supplementary Information

**Additional file 1: Table S1. **PCR amplification system.

## Data Availability

All the submissions have been made to the GenBank. All NCBI GenBank accession numbers are as follows, *Microbacterium trichothecenolyticum* strain TJ-1-57(MF196242), *Microbacterium esteraromaticum* strain TJ-1-56(MF196241), *Arthrobacter pascens* strain BJX:3(MK961252), *Pseudonocardia carboxydivorans* strain BJX:8(MK961253), *Ochrobactrum anthropi* strain TJ-1-58(MF196243), Uncultured fungus clone BJZ-5 (MN309851.1), Uncultured fungus isolate BJZ-1 (MN309844.1), Uncultured fungus clone BJZ-22 (MN309846.1), Uncultured fungus clone BJZ-29(MN309849.1), Uncultured fungus clone BJZ-18 (MN309854.1), Uncultured fungus clone BJZ-21 (MN309845.1), Uncultured fungus clone BJZ-24(MN309847.1), Uncultured fungus clone BJZ-2 (MN309850.1), Uncultured fungus clone BJZ-8 (MN309853.1), Uncultured fungus clone BJZ-26 (MN309848.1), and Uncultured fungus clone BJZ-7 (MN309852.1).
