# Supplementary material for: Microbial degradation and community structure analysis of hydroxyl-terminated polybutadiene (HTPB)
Source: AMB Express. 2021 Dec 27;11:180. doi: 10.1186/s13568-021-01334-1 (PMC8712286; doi:10.1186/s13568-021-01334-1)
Supplement: Supplementary file 1 — Additional file 1: Table S1. PCR amplification system. [file 13568_2021_1334_MOESM1_ESM.docx]

**Table S1**

| Reagent name | Volume |
| --- | --- |
| 10×Taq Buffer | 2.50 μL |
| dNTP Mixture (2.5 mmol/L) | 0.50 μL |
| MgCl_2_(25 mM) | 1.50 μL |
| P_0_ primer(10 umol) | 0.50 μL |
| P_6_ primer(10 umol) | 0.50 μL |
| Taq enzyme（5 U/μL） | 0.30 μL |
| Template (total genomic DNA) | Bacteria/2 μL |
| Sterile water | 19.20 μL/17.20 μL |
| Total capacity | 25.0 μL |
